# Neurons generated from carcinoma stem cells support cancer progression

**DOI:** 10.1038/sigtrans.2016.36

**Published:** 2017-01-06

**Authors:** Ran Lu, Chuanwen Fan, Wenqi Shangguan, Yuan Liu, Yu Li, Yanna Shang, Dongqin Yin, Shengliang Zhang, Qiaorong Huang, Xue Li, Wentong Meng, Hong Xu, Zongguang Zhou, Jiankun Hu, Weimin Li, Lunxu Liu, Xianming Mo

**Affiliations:** 1 Laboratory of Stem Cell Biology, State Key Laboratory of Biotherapy/Collaborative Innovation Center of Biotherapy, West China Hospital, Sichuan University, Chengdu, China; 2 Institute of Digestive Surgery, State Key Laboratory of Biotherapy/Collaborative Innovation Center of Biotherapy, West China Hospital, Sichuan University, Chengdu, China; 3 Department of Gastrointestinal Surgery, State Key Laboratory of Biotherapy/Collaborative Innovation Center of Biotherapy, West China Hospital, Sichuan University, Chengdu, China; 4 Laboratory of Gastric Cancer, State Key Laboratory of Biotherapy/Collaborative Innovation Center of Biotherapy, West China Hospital, Sichuan University, Chengdu, China; 5 Department of Respiratory and Critical Care Medicine, State Key Laboratory of Biotherapy/Collaborative Innovation Center of Biotherapy, West China Hospital, Sichuan University, Chengdu, China; 6 Department of Thoracic Surgery, State Key Laboratory of Biotherapy/Collaborative Innovation Center of Biotherapy, West China hospital, Sichuan University, Chengdu, China

## Abstract

Recent evidences show that nervous system acts as a crucial part of cancer microenvironment. Infiltration of nerve fibers into cancer microenvironment has an important active role in cancer progression. The stimulations of both cancer growth and metastasis by members of nervous system such as neurons and glial cells have been demonstrated. However, how the nervous system is built in cancer is largely unknown. Here we show that a fraction of cancer stem cells (CSCs) derived from patients with gastric carcinoma and colorectal carcinoma are capable of producing neurons that are involved in tumor neurogenesis and tumor growth. Cancer stem cell monoclone derived from a single cancer stem cell was able to generate neurons including sympathetic and parasympathetic neurons to take part in the nervous system in cancer tissues. Knocking down the neural cell generating capability of the human CSCs inhibited the growth of xenograft tumors in mouse model. Our data demonstrate that human CSCs are able to produce one of most important components in the cancer microenvironment that are required for cancer development and progression.

## Introduction

The observations on the association between cancer and nervous system can be traced back to early years of ninteenth century.^1^ Nerves have an important role in tumor growth, cancer invasion and even metastasis and are considered to be components of cancer microenvironment.^2^ A process termed perineural invasion that cancer cells can grow around and eventually invade existing nerves has been observed in many kinds of cancers and is generally associated with poor survival and prognosis.^3–6^ Cancer cells can attract nerve fibers and stimulate nerve outgrowth by secreting neurotrophic factors.^7,8^ Conversely, nerve fibers can infiltrate tumor microenvironment and stimulate tumor growth and cancer cell dissemination.^9^ Recent studies have revealed that autonomic nerves are necessary in all phases of prostate cancer development.^10^ Surgical and pharmacological ablation of nerves in the stomach of mice with gastric cancer showed significant inhibition effects on tumorigenesis, tumor development and a promotion effect on chemotherapy.^11^ Targeting cancer neurogenesis may be promising in the development of new cancer treatment. However, the key drivers of neuron outgrowth in tumors have not been identified and how the nervous system built in cancer tissues is largely unknown. Here we tested the potential of cancer stem cell to differentiate into neurons and the capacity of cancer cells to participate in the process of cancer neurogenesis.

## Materials and methods

### Cancer stem cell isolation and culture

Tumor surgical specimens were collected in accordance with a protocol approved by the West China Hospital of Sichuan University Institutional Ethics Committee. Informed consent was obtained from all patients. Colorectal cancer stem cell and gastric cancer stem cell were derived from colorectal and gastric adenocarcinoma tumors and functionally validated as described previously.^12,13^ In *in vitro* differentiation assays, cells were seeded on coverclips pretreated with Matrigel Matrix Growth factor reduced (Corning, Bedford, MA, USA) and induced to differentiate in Dulbecco's modified Eagle's medium medium containing 2% fetal bovine serum and B27 (Thermo) with vitamin A. Following shRNAs were used and the corresponding lentiviruses were from Genepharma (Shanghai, China): Microtubule Associated Protein 2 (MAP2) shRNA1 ( 5′-GCGCCAATGGATTCCCATACA-3′), MAP2 shRNA2 (5′- GCACCTGACCTTCCTGAAATG-3′) and control shRNA ( 5′-TTCTCCGAACGTGTCACGT-3′).

### MAP2 promoter-driven expression of ZsGreen

Human MAP2 promoter (1487 bp)^14^ was cloned by PCR and confirmed by sequencing. The promoter was inserted into pLVX-IRES-ZsGreen1-EF-puro lentiviral vector to replace the original CMV promoter. Lentiviruses were produced and tittered as described elsewhere.^15^

### Immunofluorescent staining

Coverclips and frozen sections were fixed with 4% paraformaldehyde or methanol/acetone. In experiments that paraformaldehyde was used for fixation permeablization was performed with 0.5 to 1% Trion X-100. After blocked with 5% bovine serum albumin in PBS-Tween for 1 h, fixed cells or frozen sections were incubated with primary antibodies overnight at 4 °C in PBS-Tween with 3% bovine serum albumin. The primary antibodies used were: Beta-3-tublin (Chicken, Novus, Littleton, CO, USA nb100-1612), NuMA (Rabbit, Abcam, Cambridge, MA, USA ab84680), NuMA (Goat, Santa-Cruz, Dallas, TX, USA sc-18557), MAP2 (Rabbit, Santa-Cruz sc-20172), CDX2 (Mouse, Origene, Beijing, China TA500251), CK20 (Rabbit, Abcam ab-76126), TH (Chicken, Abnova, Taipei City, China PAB29094), Vacht (Rabbit, Sigma, St Louis, MO, USA SAB4200559), SV2 (Goat, Santa-Cruz sc-11936), Synapsin I (Rabbit, Abcam ab-64581). Secondary antibodies specific to the appropriate species were used (1:500; Jackson ImmunoResearch Laboratories, West Grove, PA, USA & Thermo-Fisher, Waltham, MA, USA). All immunofluorescent staining results of *in vitro* cultured cell shown in this article were replicated for more than five times. All of the immunofluorescent staining results of frozen sections of xenografts were replicated more than 3 times.

### Animal experiment

Animal experiments were performed as described previously.^12,13^ In detail, male or female nude mice (BALB/C strain), 4–6 weeks old, were purchased from the Beijing Experimental Animal Center of the Chinese Academy of Sciences (Beijing, China). Mice in this study were housed under pathogen-free conditions, and all animal studies were carried out according to the animal protocol approved by the Sichuan University Institutional Animal Care and Use Committee. In all experiments, a small aliquot of cells was set aside to confirm cell counts and viability using conventional techniques (that is, Trypan blue exclusion) or 7-AAD staining. Once cell counts and viability were confirmed, cells were diluted to appropriate injection doses for intraperitoneal or subcutaneous injection. In intraperitoneal injection, cells were suspended in PBS and injected into mice intraperitonealy. In subcutaneous injection, cells were mixed with Matrigel (Corning) at a 1:1 ratio, and injected subcutaneously in nude mice on the ventral wall. No randomization or blinding techniques were applied in this study. Injected mice were killed when the established criteria for end-stage diseases were reached.

### RNA extraction and real-time quantitative PCR

Total RNA of cells was extracted with a Trizol reagent kit (Takara Biotechnology Co., Ltd. Dalian, China) according to the manufacturer’s protocol. Subsequently, reverse-transcription of RNA by PCR was performed using a Takara SYBR real-time PCR kit for target gene.

### Statistics analysis

Data were statistically analyzed. For two experimental comparisons, a two-tailed unpaired Student’s *t*-test was used. The difference between experimental groups was assessed by one-way analysis of variance testing. When cells were used for experiments, three replicates per treatment were chosen as an initial samplesize. All *n* values defined in the legends refer to biological replicates. If technical failures such as inadequate subcutaneous or intraperitoneal injection occurred before collection, those samples were excluded from the final analysis.

### Data availability

The authors declare that the data supporting the findings of this study are available within the paper.

## Results

### Neural cells with human-specific marker appear in tumor xenografts in mice

**Figure 1:**
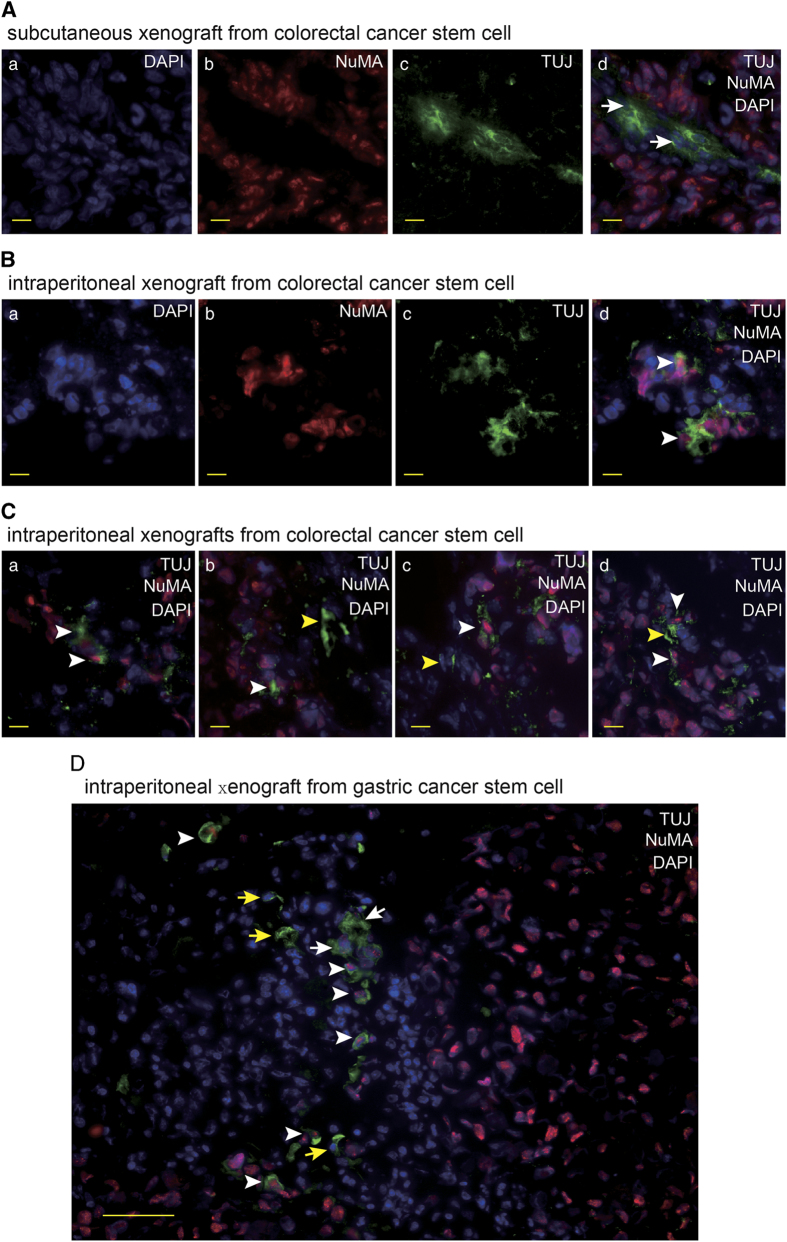
The neural cells carry human markers in the xenografts generated from human gastric and colorectal cancer stem cells. (**A**) IF staining of beta-3-tublin (TUJ) and NuMA in frozen section of subcutaneous tumor xenograft derived from human colorectal cancer stem cell with the scale bar representing 10 μm. Arrows indicate mouse originated TUJ-expressing cells. (**B**, **C**) IF staining of TUJ and NuMA in frozen section of intraperitoneal tumor xenograft derived from human colorectal cancer stem cell with the scale bar representing 10 μm. Arrows indicate TUJ-positive cells with human origin (**B**) and adjacent TUJ-positive cells with human origin (white arrows) and mouse origin (yellow arrows), respectively (**C**). (**D**) IF staining of TUJ and NuMA in frozen section of intraperitoneal tumor xenograft derived from human gastric cancer stem cell with the scale bar representing 50 μm. Arrows indicate adjacent TUJ-positive cells with human origin (white arrows) and mouse origin (yellow arrows), respectively.

In order to determine the involvement of neural system in the tumors that were generated from human cancer stem cells (CSC) *in vivo*, we transplanted the CSCs that were previously isolated from the patients with gastric and colorectal carcinoma^12,13^ into nude mice via subcutaneous and intraperitoneal injections to produce human cancer xenografts. Tumor innervations were observed. The neural staining showed that many ganglia generated from mouse nerve system infiltrated into the interstitial tissues in the tumor masses (Figure 1a). The IF staining of frozen sections of intraperitoneal tumor xenografts showed a significant fraction of neural cells carried the human-cell-specific nuclear antigen nuclear mitotic apparatus protein (NuMA).^16^ The neural cells with human origin were distributed in the ganglia closed to tumor masses and in tumor tissues (Figures 1b–d). In addition, the sections of subcutaneous xenografts were detected fewer neural cells with human markers in the ganglia, in comparison with intraperitoneal tumor xenografts. The results suggest that gastric CSCs and colorectal CSCs may have the capacity to differentiate into neural cells to generate nervous system in tumor tissues in tumor xenografts.

### CSC monoclones derived from single human cancer stem cells can generate neural cells *in vitro*

**Figure 2:**
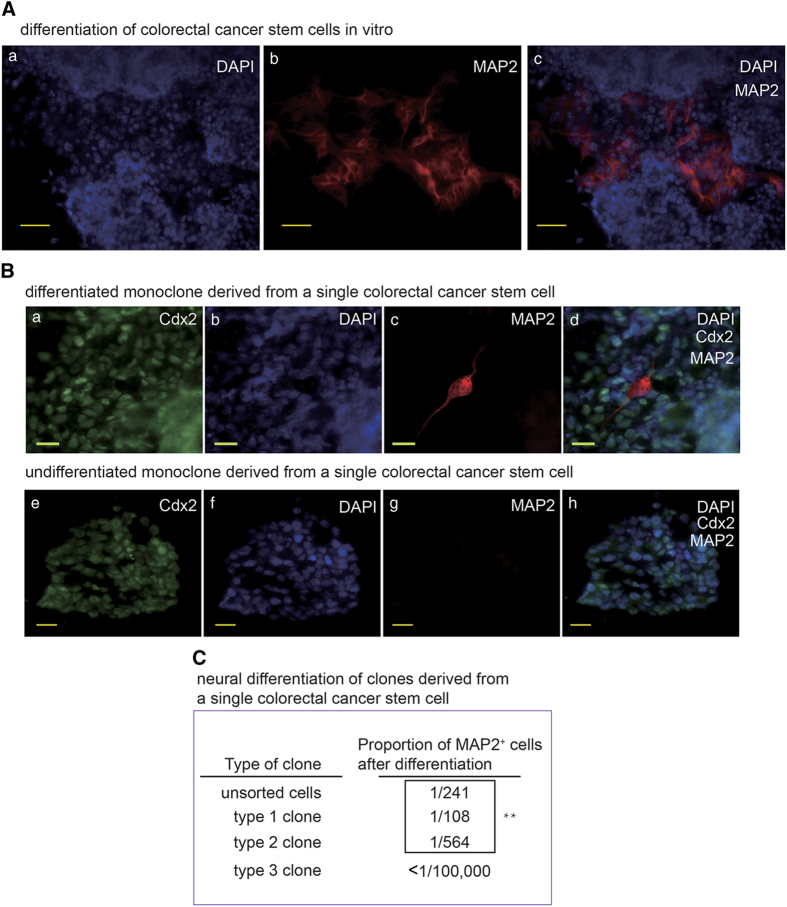
Monoclonal human gastric and colorectal cancer stem cells produce neural cells. (**A**) IF staining of MAP2 in *in vitro* differentiated colorectal cancer stem cell with the scale bar representing 50 μm. (**B**) a–d: IF staining of colorectal cancer marker Caudal Type Homeobox 2 (CDX2) and MAP2 in *in vitro* differentiated colorectal cancer stem cell with the scale bar representing 20 μm. Cells were from a clone derived from a single colorectal cancer stem cell. e–h: IF staining of colorectal cancer marker CDX2 and MAP2 in undifferentiated colorectal cancer stem cell sphere frozen section with the scale bar representing 20 μm. Cells were from a clone derived from a single colorectal cancer stem cell. (**C**) Counting results of proportion of MAP2^+^ cell in unsorted colorectal cancer stem cells and 3 colorectal cancer stem cell derived monoclones *in vitro* differentiated for 10 days. Positive cell numbers and total cell numbers from five random fields (except for type 3 monoclone because this type almost didn’t produce any MAP2^+^ cell.) were quantified with a Carl Zeiss Axio Scope.A1 microscope under ×20 magnification, by counting cells on 30–50% of one field area and extrapolate to 100% of the field. **P<0.01 by one-way analysis of variance test.

To determine the neural differentiation potential of gastric and colorectal CSCs, Cultured gastric and colorectal CSCs were induced to differentiate for more than 7 days. Neural cell foci among the epithelial cancer cells were detected in IF staining of differentiated CSCs (Figure 2A). To verify the capacity of CSCs to differentiate into neurons, gastric and colorectal CSC monoclones were generated from single cancer stem cells. The CSC monoclones were then differentiated with serum induction. IF staining demonstrated that the CSC monoclones did generate neural cells. We also examined the undifferentiated CSC spheres and did not observe neural cells (Figure 2B and data not shown). Then, the gastric and colorectal CSC monoclones were induced to differentiate by medium optimized for neural stem cell to produce neuron for further examination of their differentiation potential. The capabilities to produce neural cells of different CSC monoclones are different (Figure 2C), suggesting that CSCs derived from the patient samples are heterogeneous and only a fraction of CSCs are able to give rise to neural cells. The capacity of the CSC monoclones to form tumor xenografts in nude mice was verified by subcutaneous and intraperitoneal implantation (data not shown). These data ruled out the possibility that our results were caused by cross neural cell contamination when we isolated the CSCs from the patient samples and confirmed that gastric and colorectal CSCs are able to produce neural cells.

**Figure 3:**
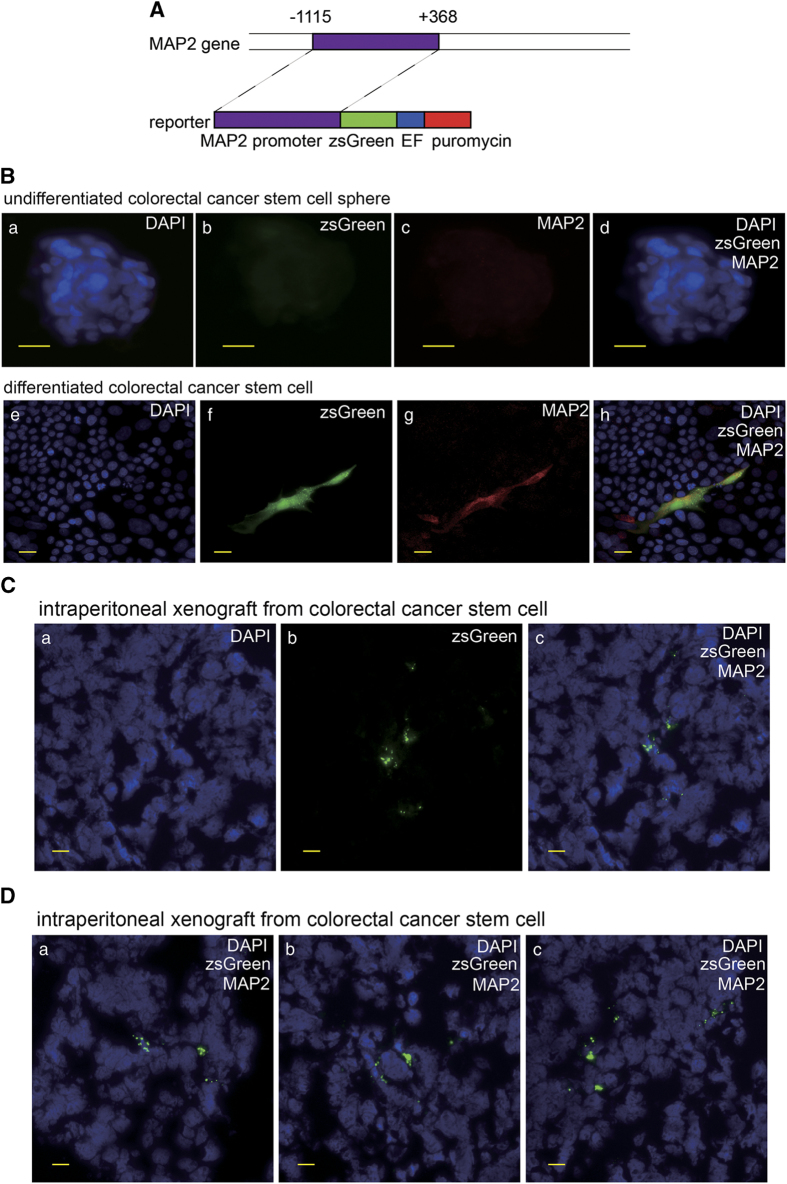
The zsGreen driven by MAP2 promoter is expressed in the differentiated cancer cells generated from human colorectal cancer stem cells. (**A**) Schematic diagram of the zsGreen reporter driven by MAP2 promoter. (**B**) a–d: zsGreen expression and IF of MAP2 in colorectal cancer stem cell sphere with the scale bar representing 20 μm. Cells carry the MAP2 promoter driving zsGreen reporter. e–h: zsGreen expression and IF of MAP2 in *in vitro* differentiated colorectal cancer stem cell carrying the MAP2 promoter driving zsGreen reporter with the scale bar representing 20 μm. (**C**) zsGreen expression detected in frozen section of intraperitoneal tumor xenograft derived from human colorectal cancer stem cell carrying MAP2 promoter driving zsGreen reporter with the scale bar representing 20 μm.

To provide further evidence validating neural differentiation capacity of the gastric and colorectal CSCs, we cloned the human MAP2 promoter and generated lentiviral construct for MAP2 promoter-driven zsGreen expression (Figure 3A) and determined the zsGreen expression of transduced CSCs *in vitro* and in tumor xenografts in mice. IF staining of the cultured differentiated CSC showed the cells expressing zsGreen were positive for MAP2 (Figure 3B). In addition, zsGreen-positive cells in frozen section of mouse celiac xenograft were detected (Figure 3C). These results provided evidence supporting our discovery that the gastric and colorectal CSCs carry neural differentiation potential.

### Human cancer stem cells can produce functional neurons

**Figure 4:**
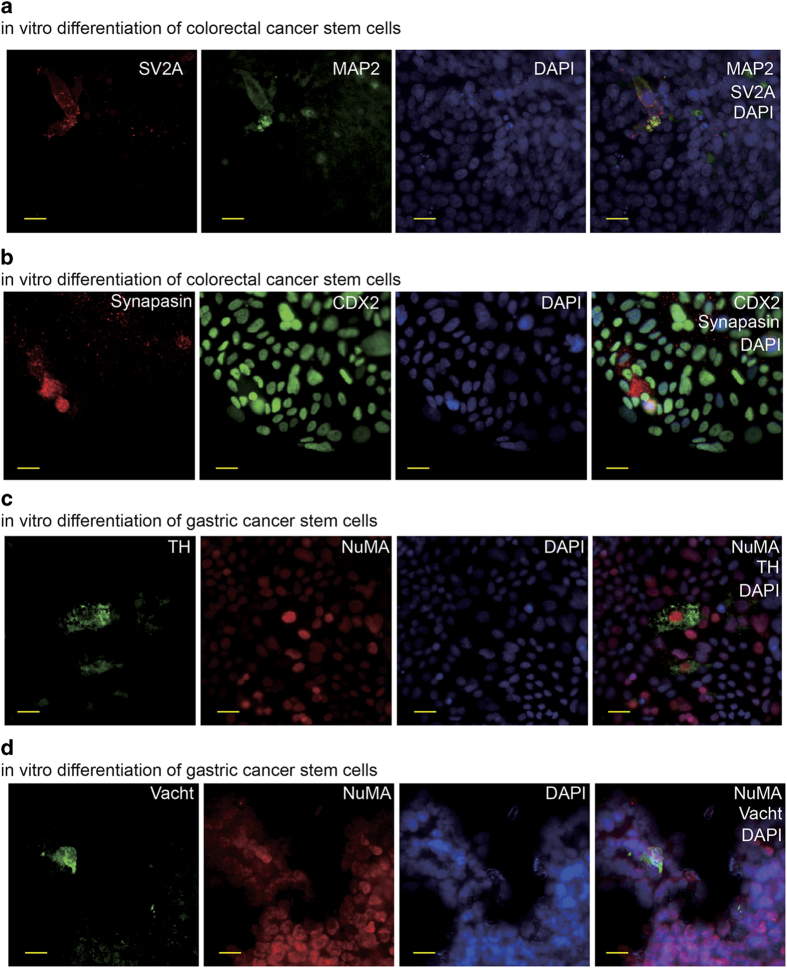
The neurons are generated from human gastric and colorectal cancer stem cells. (**a**) IF staining of synapse marker Synaptic Vesicle Protein 2A (SV2A) and neuron marker MAP2 in *in vitro* differentiated human colorectal cancer stem cell with the scale bar representing 20 μm. (**b**) IF staining of synapse marker Synapsin and neuron marker MAP2 in *in vitro* differentiated human colorectal cancer stem cell with the scale bar representing 20 μm. (**c**) IF staining of MAP2 and sympathetic neuron marker TH in *in vitro* differentiated human gastric cancer stem cell with the scale bar representing 20 μm. (**d**) IF staining of MAP2 and parasympathetic neuron marker Vacht in *in vitro* differentiated human gastric cancer stem cell with the scale bar representing 20 μm.

**Figure 5:**
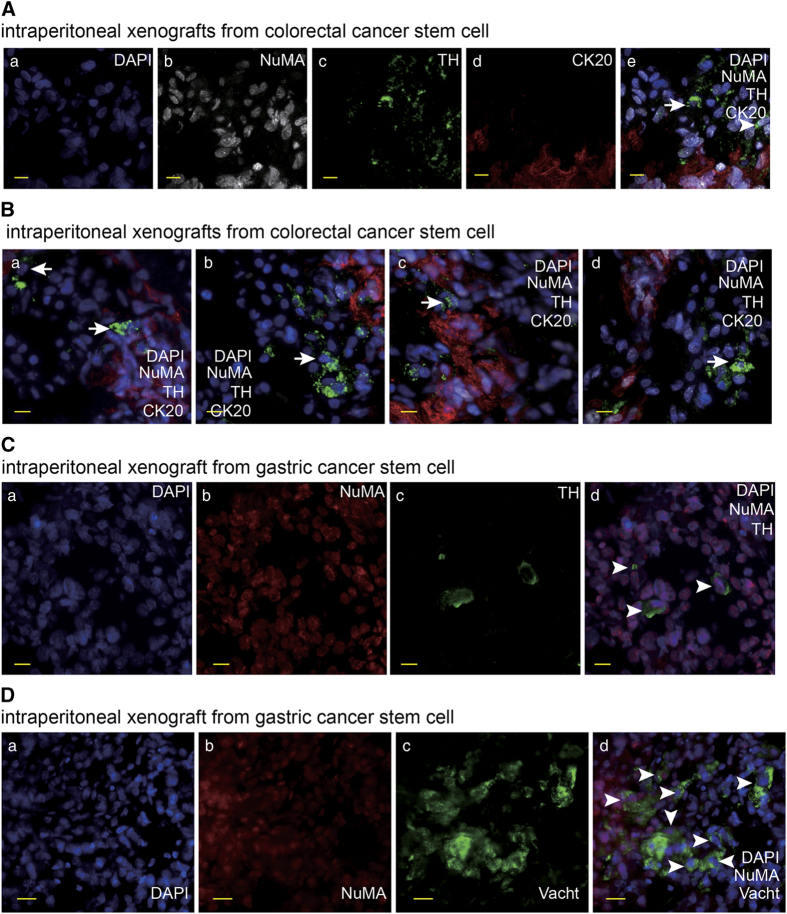
The autonomic neurons are produced from human gastric and colorectal cancer stem cells in xenografts. (**A**, **B**) IF staining of NuMA, TH and colorectal cancer marker CK20 in frozen section of intraperitoneal tumor xenograft derived from human colorectal cancer stem cell with the scale bar representing 20 μm. Arrows indicate TH-positive cells with human origin. (**C**) IF staining of NuMA and TH in frozen section of intraperitoneal tumor xenograft derived from human gastric cancer stem cell with the scale bar representing 10 μm. Arrows indicate TH-positive cells with human origin. (**D**) IF staining of NuMA and Vacht in frozen section of intraperitoneal tumor xenograft derived from human gastric cancer stem cell with the scale bar representing 20 μm. Arrows indicate Vacht-positive cells with human origin.

We examined the potential to form synapses, one of the most important structures of functional neurons,^17^ of the neural cells derived from the gastric and colorectal CSCs. A fraction of differentiated CSCs were stained positive for synapse markers (Figures 4a and b), suggesting that the human CSCs are able to produce functional neurons. Previous evidence has demonstrated that the autonomic neural system contributes to cancer progression.^9,10,18–20^ Therefore, we proceeded to determine whether there were autonomic neurons among the neural cells derived from human CSCs. The results showed that a small number of differentiated colorectal and gastric CSCs were positive for sympathetic neuron marker tyrosine hydroxylase (TH) (Figure 4c and data not shown). It was reported that cultured colorectal cancer cell lines can produce parasympathetic transmitter acetylcholine.^21^ For this reason, we did not try to detect parasympathetic neurons in differentiated colorectal CSCs. However, we tested the potential of gastric cancer stem cells to produce parasympathetic neurons. Cells expressing parasympathetic neuron marker vesicular acetylcholine transporter (VaChT) were observed among differentiated gastric CSCs (Figure 4d). Next, we searched for autonomic neurons in frozen sections of xenografts derived from human gastric and colorectal CSCs. Human originated sympathetic neurons were detected in frozen sections of mouse celiac tumor xenografts derived from human gastric and colorectal CSCs (Figures 5a–c). The results were further confirmed by the xenografts generated from the monoclones derived from single colorectal CSCs (data not shown). In addition, the human originated parasympathetic neurons in mouse celiac tumor xenograft derived from human gastric cancer stem cell were also detected (Figure 5d). Taken together, these data demonstrate that human gastric and colorectal CSCs are capable of producing neurons, especially autonomic neurons when they generate tumor masses.

### Knocking down the neural generating ability of human cancer stem cells decreases the tumor growth *in vivo*

**Figure 6:**
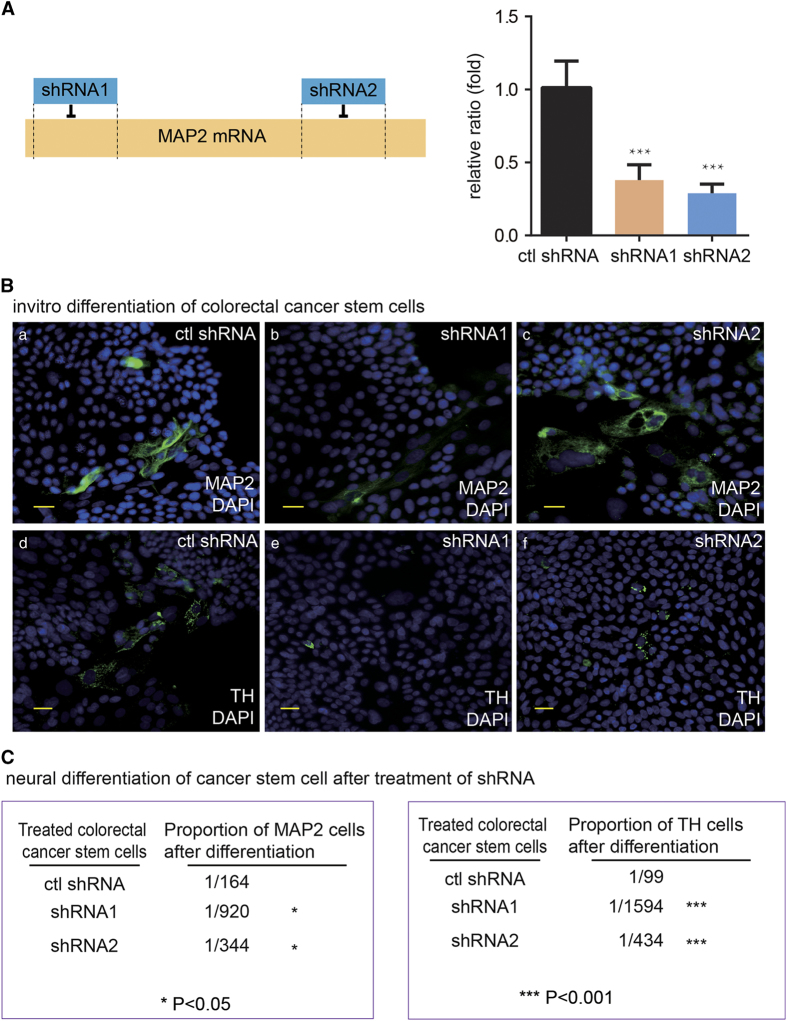
The knocking down of MAP2 expression significantly decreased the generation of neural cells from human gastric and colorectal cancer stem cells. (**A**) Schematic diagram of 2 MAP2-targeting shRNAs loci in MAP2 gene and shRNA efficiency verified by qRT-PCR. Value represent mean±s.d. of independent biological triplicates. ***P<0.001 by Student’s *t*-test. (**B**) a–c: IF staining of MAP2 in *in vitro* differentiated colorectal cancer stem cell expressing control shRNA, MAP2 shRNA1 and MAP2 shRNA2, respectively, with the scale bar representing 20 μm. Cells were from a single colorectal cancer stem cell. d–f: IF staining of TH in *in vitro* differentiated colorectal cancer stem cell expressing control shRNA, MAP2 shRNA1 and MAP2 shRNA2, respectively. Cells were from a single colorectal cancer stem cell. (**C**) Counting results of proportion of MAP2^+^ cell and TH^+^ cell in colorectal cancer stem cells expressing control shRNA, MAP2 shRNA1 and MAP2 shRNA2, respectively. Positive cell numbers and total cell numbers from five random fields were quantified with a Carl Zeiss Axio Scope.A1 microscope under ×20 magnification, by counting cells on 30–50% of one field area and extrapolate to 100% of the field. Mean values of calculated cell proportion were shown in the figure. *P<0.05, ***P<0.001 by Student’s *t*-test.

**Figure 7:**
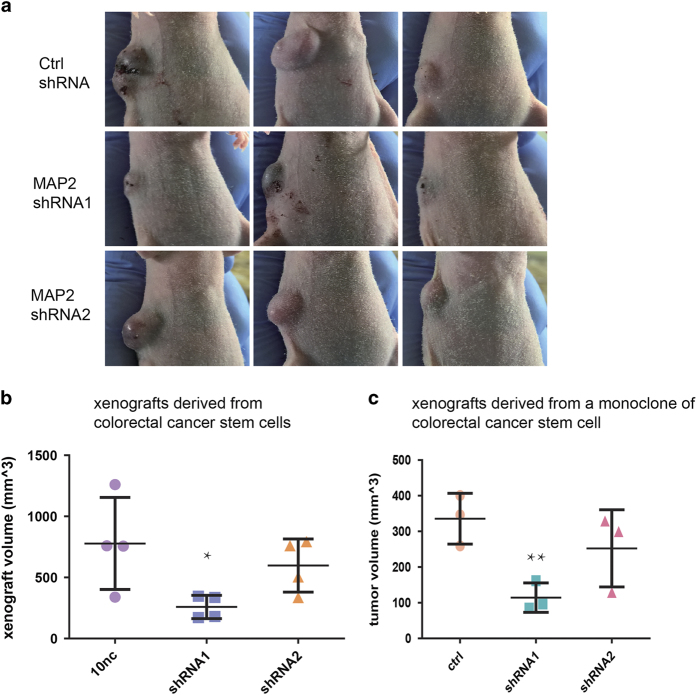
The knocking down MAP2 expression significantly reduced the growth of xenografts generated by human colorectal cancer stem cells. (**a**) Nude mice with subcutaneous tumor xenograft derived from human colorectal cancer stem cell expressing control shRNA, MAP2 shRNA1 and MAP2 shRNA2 respectively. (**b**) Comparison of tumor xenograft volumes of mouse subcutaneous tumor xenograft derived from human colorectal cancer stem cell. Human colorectal cancer stem cell expressing MAP2 shRNA1, MAP2 shRNA2 and control shRNA respectively were subcutaneously injected into nude mice at 8×10^4^ cells per mouse. Diameters of tumor xenograft were measured 29 days after injection of cell. (*n*=4 mice per group). This experiment was conducted 3 times with similar results. (**c**) Comparison of tumor xenograft volumes of mouse subcutaneous tumor xenograft derived from a clone from a human colorectal cancer stem cell. Monoclonal human colorectal cancer stem cell expressing MAP2 shRNA1, MAP2 shRNA2 and control shRNA, respectively, were subcutaneously injected into nude mice at 8×10^4^ cells per mouse. Diameters of tumor xenograft were measured 26 days after injection of cell. (*n*=3 mice per group). **P<0.01 by Student’s *t*-test. This experiment was conducted twice and the results were similar. Values in **b** and **c** represent mean±s.d. of indicated number of mice.

Recent data have revealed that the crosstalks between cancer cells and nervous system are critical for tumorigenesis, tumor growth and tumor metastasis.^2,22,23^ After knowing that the human CSCs were able to produce neurons, we speculated that CSC derived neurons may have the ability to promote tumor progression. Accordingly, we ablated the neural generating capabilities of the human CSCs by MAP2 RNAi (5′ end RNAi: shRNA1 and 3′ end RNAi: shRNA2; Figure 6A–C) to explore the involvement of neurons derived from the human CSCs in tumor progression. The detection of MAP2 mRNA showed that both 5′ end and 3′ end shRNAs markedly decreased the level of MAP2 mRNA. The ethynyl deoxyuridine incorporation of the CSCs were not affected by MAP2 knock-down, suggesting that the CSC neural differentiation potential are not related to the proliferation *in vitro* (data not shown). We also did not detect increased apoptosis or autophagy in *in vitro* differentiated cells after knocking down MAP2. In contrast, the capacity of colorectal cancer stem cells to give rise to neural cells and the TH-positive cells was remarkably reduced by MAP2 knock-down (Figure 6B and C). The shRNA1 was more effective in reducing the neural differentiation capacity of CSCs in culture. Consistently, the tumorigenesis capacity of the human CSCs and the tumor growth in nude mice were markedly reduced by MAP2 shRNA1 and slightly knocked down by MAP2 shRNA2 (Figure 7). The results demonstrated that the human CSC derived neural cells have an important role in tumorigenesis and promote the tumor growth.

## Discussion

The supporting tissues in tumor microenvironment, including blood vessels, connection tissues and fibroblast, are indispensable for all the steps, such as tumorigenesis, growth, progression and metastasis, of cancer development in human.^2,17,24,25^ Recent studies have led to the conclusion that nerves are an important component of cancer microenvironment.^2,23,26^ Autonomic nerves especially sympathetic nerves have a significant role in the progression of cancers such as prostate cancer and gastric cancer.^9,11^ Like prostate cancer, colorectal cancer has an environment, which is rich in autonomic nerve fibers, and the presence of nerve fibers is associated with poor prognosis.^27^ Here we show that xenograft tumors derived from human colorectal cancer stem cell were infiltrated by host nude mice nerve fibers and this demonstrate that colorectal cancer cells can interact with nerve fibers just like prostate cancer cells. Our result suggests that crosstalk between neural system and cancer cells are common among solid malignant tumors. Nerve infiltration is observed in human cancer xenograft tumors formed subcutaneously and intraperitonealy, suggesting that the crosstalk between cancer cells and nerve fibers can happen in different tumor initiation environment.

As shown here, both human gastric and colorectal cancer stem cells are able to give rise to neurons when transplanted intraperitonealy in nude mice. This capacity has also been verified *in vitro* and has been confirmed both *in vivo* and *in vitro* by monoclonal cancer stem cells. Cancer stem cells are known to be able to give rise to component of tumor microenvironment such as endothelial cells and pericytes.^28–30^ Our finding extends the understanding about differentiation capability of cancer stem cell. The crosstalk between cancer and nervous system has been known for a long time and recent studies are focused on the reciprocal effects of cancer cells and nerve fibers. Neurogenesis in tumors is attributed to the attractive effect of cancer cells to normal nerve fibers via the secretion of signal molecules and neurotrophic factors. Here we demonstrate that cancer stem cells have the capacity to directly contribute to the neurogenesis in tumors. The involvement of cancer stem cells in the formation and activity of cancer microenvironment is beyond previous understanding.

We also identified that when the isolated CSCs are subcultured for >30 passages, they will gradually lose the neural differentiation capability (data not shown). Furthermore, we initiated to test the cancer stem cells derived from the patients with lung adenocarcinoma. The preliminary results showed that lung cancer stem cells also generated neural cells (data not shown).

Previous data suggested that β-blockers may help to improve survival and prognosis of cancer patients.^31–35^ Recent findings about nerve-cancer crosstalk have raised the possibility that using drugs targeting nervous system and denervation operation in combination with other therapies could be a promising approach in cancer treatment. Our result that attenuating the capacity of cancer stem cell to give rise to neurons suppressed the growth of xenograft tumor supports this possibility. The capacity of cancer stem cell to transdifferentiate into components of blood vessels may be one of the resistance mechanisms to therapies targeting cancer angiogenesis.^28–30^ It is necessary to target the process of cancer stem cell differentiation into neurons or even cancer stem cells themselves to avoid the possible cancer resistance to anti-neurogenesis therapies.
